# Can *Ganoderma* Triterpenoids Exert Immunogenic Cell Death in Human Cancer Cells? A Systematic Review and Protein Network Analysis

**DOI:** 10.3390/ph18111641

**Published:** 2025-10-30

**Authors:** Jorge C. Ramírez-Gutiérrez, J. Fernando Ayala-Zavala, Heriberto Torres-Moreno, Max Vidal-Gutiérrez, Martín Esqueda

**Affiliations:** 1 Centro de Investigación en Alimentación y Desarrollo, A.C. Carretera Gustavo Enrique Astiazarán Rosas 46, La Victoria, Hermosillo 83304, Sonora, Mexico; jramirez224@estudiantes.ciad.mx (J.C.R.-G.); jayala@ciad.mx (J.F.A.-Z.); 2 Departamento de Ciencias Químico, Biológicas y Agropecuarias, Campus Caborca, Universidad de Sonora, Avenida K SN, Eleazar Ortiz, Heroica Caborca 83600, Sonora, Mexico; heriberto.torres@unison.mx; 3 Departamento de Ciencias Químico, Biológicas y Agropecuarias, Campus Navojoa, Universidad de Sonora, Lázaro Cárdenas del Río 100, Francisco Villa, Navojoa 85880, Sonora, Mexico; max.vidal@unison.mx

**Keywords:** cancer, triterpenoids, ganoderic acids, immunogenic cell death, *Ganoderma*

## Abstract

**Background**: Permanent cancer resolution requires a complete immunological response with generation of memory against malignant cells. Immunogenic cell death (ICD) achieves this by coupling cell death with the emission of damage-associated molecular patterns (DAMPs). Current cancer treatments immunosuppress the host; thus, new alternatives are needed. *Ganoderma* species produce anticancer triterpenoids (GTs); however, their mechanism remains unclear. **Objective:** This systematic review aims to provide insights into GTs’ pharmacodynamics and assess hypothetical ICD potential. **Methods:** Web of Science and PubMed databases were consulted following PRISMA guidelines. Studies from inception until 2024, reporting molecular changes associated with GTs’ anticancer effects, were considered. Nonhuman models were excluded. GTs and GTs-ICD converging molecular targets were listed and submitted to Cytoscape’s stringApp to construct protein interaction networks. Topological and enrichment analysis were performed. **Results:** A total of 204 articles were found, and 69 remained after screening. Overall anticancer effects include loss of mitochondrial membrane potential, DNA and RNA damage, autophagy, cell cycle arrest, and leukocyte activation. 136 molecular targets of GTs were identified; upregulated proteins include CHOP, PERK, p-eIF2α, and HSP70, a key DAMP. GTs and ICD share 24 molecular targets. GO:BP and KEGG enrichment analysis suggest that GTs’ anticancer effects are related to stress response, cell death regulation, and PD-L1/PD-1 checkpoint inhibition. GT-ICD enrichment converges on endoplasmic reticulum stress, unfolded protein response, and organelle membrane perforation. **Conclusions:** GTs exhibit polypharmacological anticancer effects, including anti-immunosuppression, upregulation of ICD-adjacent machinery, and even an increase in HSP. However, further studies are required to confirm a proper causal link between GTs’ cancer cell treatment and DAMP emission.

## 1. Introduction

Nature’s molecular repertoire is immeasurable and therefore provides scaffolds from which we can derive countless pharmacological treatments [1]. *Ganoderma* species synthesize highly oxidized lanostane-type triterpenoids through the mevalonate pathway [2]. These *Ganoderma* triterpenoids (GTs) have been extensively studied as potential therapeutic agents for the treatment of major diseases, including diabetes, heart disease, inflammatory conditions, and cancer [3]. The central subgroup of GTs that stand out are the ganoderic acids (GAs). In recent times, preclinical evidence has accumulated supporting the anticancer effects of GAs and other GTs, which have been confirmed in silico [4,5,6,7,8], in vitro [9,10,11,12,13], and in vivo with non-human models [14,15,16,17].

GAs and other GTs have demonstrated direct cytotoxic effects against a wide range of human cancer cell types, including but not limited to breast [18], prostate [10], lung [19,20], cervical [21], and colon [22]. Additionally, these compounds have demonstrated immune-modulatory effects in vitro, enhancing HLA class II antigen presentation and CD4^+^ cell activation [23,24], as well as in vivo, with improved cytokine profiles, enhanced NK cell function, and CD8^+^ cell activation [24,25,26,27]. However, much remains to be elucidated regarding the anticancer effects of GTs, namely the precise mechanisms and pharmacodynamics that lead to cancer cell inhibition.

Beyond just remission, permanent defeat of a cancerous process within the organism requires sustained inhibition of malignant cells. A robust and adaptive immune response is necessary to achieve this without further administration of exogenous agents, leading to the development of memory against tumour-derived antigens [28]. Cancer cells emit neoepitopes that are recognized by naive T cells [29]. Although cancer is often associated with proliferation, a high degree of cancer cell death also ensues, which should provide ample antigen availability. Why, then, is proper immunity against one’s cancer seldom observed? A complete answer is beyond the scope of this article, but a crucial concept to remember is that antigenicity is just one step for actual immunogenicity. A high degree of adjuvanticity is also required to bridge the gap between cancer cell antigens and the antitumoral adaptive response [30]. This is crucial for the middlemen of adaptive immune activation: the dendritic cells (DCs) [31,32].

The foundation of this bridge is built upon the induction of cell death, resulting in the emission of damage-associated molecular patterns (DAMPs), which are intracellular molecules with diverse physiological functions that become exposed on the cell surface or secreted [33]. Notable examples include calreticulin (CALR), heat shock proteins 70 and 90 (HSP70/90), high-mobility group box 1 (HMGB1), annexin A1 (ANXA1), and ATP (Table 1).

These DAMPs are recognized by antigen-presenting cells, including DCs, through their pattern recognition receptors (PRRs) [80]. Coupling phagocytosis of cell remains and PRR engagement results in activation and maturation of DCs, which then migrate to the nearest draining lymph node (Table 1). Upon arrival, their now-activated antigen-processing machinery loads antigens on major histocompatibility complex (MHC)-I and MHC-II molecules for T-cell presentation, generating an adaptive immune response [81].

The pipeline of cell death, with DAMP emission to DC maturation and migration, to T-cell priming and activation, constitutes the fundamental core concept of immunogenic cell death (ICD) [82,83]. Some authors argue that research progress in this field is expected to become the mecca of modern cancer control [84].

This review will focus on two main objectives. First, to consolidate the evidence of *Ganoderma* triterpenoids (GTs) against human cancer cells, enlisting molecular changes reported to be associated with such anticancer effects. Second, to elucidate whether such molecular changes indicate a possible connection with the DAMP emission machinery associated with immunogenic cell death. Building such a theoretical framework could incentivize further in-depth mechanistic research on GTs, with the ultimate goal of developing novel natural product-based immunotherapies that could lead to improved and more durable outcomes in cancer treatment.

## 2. Materials and Methods

### 2.1. Molecular Literature of Ganoderma Triterpenoids (GTs) vs. Human Cancer

This systematic review adheres to the guidelines in the Preferred Reporting Items for Systematic Reviews and Meta-Analyses (PRISMA) [85]. The electronic databases Web of Science and PubMed were consulted for literature about *Ganoderma* triterpenoids (GTs) in the context of antiproliferative effects against human cancer cells.

#### 2.1.1. Inclusion Criteria

Original research articles from inception until the end of December 2024 were considered; reviews and other types of publications were excluded from the initial search via database platform filters. Keywords like “cancer” or “tumour” plus phrases like “ganoderic acid” and “lanostane terpenoids” were used. Negative Boolean modifiers were used against murine, mouse, or viral models. A complete breakdown of the searched keywords and key phrases utilized is available in the Supplementary File S1 “sd1_-_search_strategy.odt”. Each retrieved publication was assessed in its full text when available by two reviewers (J.C.R.-G., and M.E.), with any disagreements resolved through mediation by a third member.

#### 2.1.2. Exclusion Criteria

Any article with the following: (a) results that do not report protein changes at the transcriptional (mRNA), translational (e.g., Western blots), or interactional level (either in silico or in vitro assays); (b) if the anticancer effect of the *Ganoderma* compound(s) was observed in non-human cancer cell lines (e.g., RIN-5F); (c) research with no mention of triterpenoids within the *Ganoderma* preparation utilized for the experiments; (d) articles in which the main research objective is unrelated to cancer. A complete list of excluded literature with the exclusion motive(s) is provided in the Supplementary File S2 “sd2 excluded_articles_and_reasons_for_exclusion.ods”.

### 2.2. Protein Libraries

#### 2.2.1. GT Targets Against Human Cancer Cells

Once the final inclusion list of articles was achieved, every publication was manually assessed for any protein or mRNA reported to change after either in vitro or in silico assays utilizing GTs in the context of anticancer treatment. A list of protein names with their corresponding UniProt [86] accession numbers was made.

#### 2.2.2. Molecular Participants of ICD

A list of proteins involved in the phenomenon of immunogenic cell death (ICD) was constructed by consulting the Online Mendelian Inheritance in Man [87] and GeneCards [88] databases with the key phrase “immunogenic cell death”. Additional targets were extracted by submitting the seminal ICD publications of “Consensus guidelines for the detection of immunogenic cell death” [89] and “ Consensus guidelines for the definition, detection, and interpretation of immunogenic cell death” [82] into the STRING database [90], from which nodes were retrieved as a list of proteins. After merging all lists, duplicates were removed. Search retrieval of ICD participants is provided in the Supplementary File S3 “sd3 icd_-_retrieval.ods”.

#### 2.2.3. Intersection Between GTs Targets and ICD

The list of molecular targets affected by GTs and the list of ICD proteins previously obtained were then input to the Venny online tool for the construction of a Venn diagram [91]. Intersecting targets were retrieved as an additional list, deemed as the molecular bridge connecting GTs-ICD, which was utilized for subsequent analyses.

### 2.3. Protein–Protein Interaction (PPI) Network Construction

The list of GTs’ molecular targets and the list of GT-ICD intersections were submitted to the STRING v12.0 database [90] through the Cytoscape (v3.10.3) StringApp v2.2.0 [92,93]. The following parameters were used to construct the protein interaction network: *Homo sapiens* as species, full STRING network type, high confidence cutoff point (>0.700), and no additional interactors. The Cytoscape sessions are provided as the Supplementary File S4 “sd4 134_GTs_targets.cys” and File S5 “sd5 24_GTs-ICD_targets.cys”.

### 2.4. GO and KEGG Enrichment Analyses

The obtained networks were subjected to Gene Ontology (GO) functional analysis and Kyoto Encyclopaedia of Genes and Genomes (KEGG) pathway enrichment analysis using the StringApp v2.2.0. The False Discovery Rate (FDR) was employed as the statistical testing method [94]. Because the entire human genome was used as background, a redundancy cutoff of 0.5 was imposed to remove redundant terms from the resulting enrichment analyses lists. Complete.csv tables containing the entire enrichment result values are provided in the Supplementary File S6 “sd6 enrichment_analysis_complete_tables.zip”.

### 2.5. Data Visualization

The obtained .csv tables were submitted to RStudio IDE v4.4.2 for plotting. The top 20 most significant results for each enrichment were visualized as bubble charts using the ggplot2 v3.5.1 package.

## 3. Results

### 3.1. PRISMA

**Figure 1:**
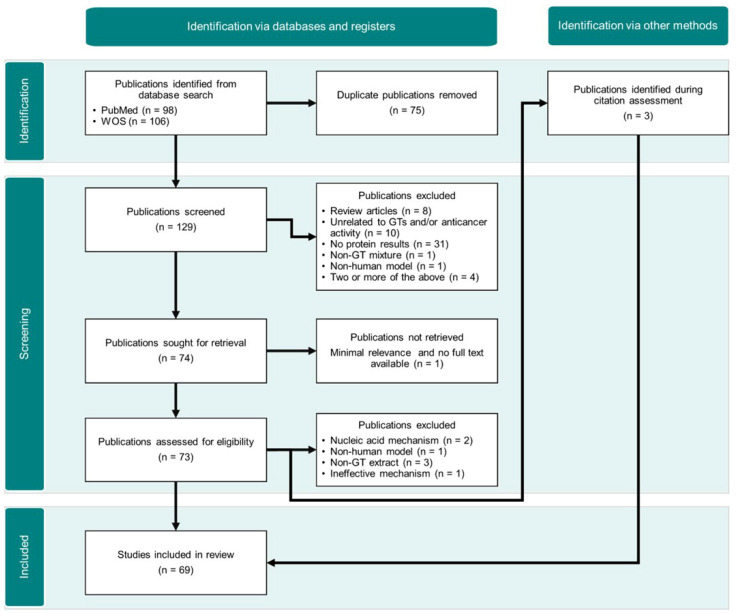
Workflow diagram based on the Preferred Reporting Items for Systematic Reviews and Meta-Analyses (PRISMA) recommendations for publication search and retrieval.

As shown in the PRISMA diagram (Figure 1), the search strategy yielded 204 publications, of which 75 duplicates were removed, amounting to 129 articles for manual screening. Abstracts and full texts (when available) were assessed, and those meeting exclusion criteria were discarded, giving 66 inclusions from the initial search. Three additional articles were manually added during the assessment because they met the inclusion criteria, despite not being identified through the initial search strategy. It is worth mentioning that, despite using the consulted platforms’ filters to avoid retrieving review articles during the search, several reviews still ended up as part of the results. Finally, 69 studies were selected, all of which explicitly report changes in protein and/or mRNA levels in human cancer cells treated with *Ganoderma* triterpenoids (GTs), or computationally calculated interactions between GTs and cancer-related proteins (Table 2).

### 3.2. Overall Findings

The entirety of the found literature was preclinical in nature. Within the studies presented in Table 1, a total of eight *Ganoderma* species were reported to produce triterpenoids with antiproliferative or anticancer potential against human cancer cells, *G. lucidum* being the most widely mentioned. *Ganoderma* triterpenoids (GTs) exerted antiproliferative and cytotoxic effects against cancer cell lines from every major organ. GTs utilized for such in vitro assays ranged from triterpenoid-enriched extracts to isolated and purified compounds. From the latter group, the majority of molecules belong to the family known as ganoderic acids (GAs), and the most studied isotypes were GA-A, GA-DM, GA-Me, and GA-T. Research mainly targeted breast, lung, and cervical-uterine tissues; the cell lines used most were HepG2, HeLa, and MDA-MB-231. Notably, eight studies also employed non-cancerous cells to test selective cytotoxicity/antiproliferation, and GTs were found to be less toxic against the ARPE-19 [4], HEK293 [97], and LO2 [97,117,119] cell lines. GTs were also less toxic against human non-cancerous cells isolated ex vivo, including healthy neurons [9], peripheral blood mononuclear cells [127], and dermal fibroblasts [119]. Forty-four studies were conducted exclusively in vitro, six were in silico, and 19 employed both types of experimentation.

Amongst non-molecular cellular effects reported, cell cycle arrest was mentioned in 18 studies, particularly at the G1 phase. Additionally, GTs caused alterations in cancer cell mitochondrial function, with 12 studies showing a decreased membrane potential of such organelle (↓ΔΨm). Other phenomena reported include reduced synthesis and direct damage to DNA and mRNA, programmed cell death, endoplasmic reticulum (ER) stress, disruption of cancer’s antioxidant capabilities, and suppression of cancerous programming, such as the reversal of the Warburg effect [22] and diminished multidrug resistance [128,129].

### 3.3. Analysis of GT Protein Targets

**Figure 2:**
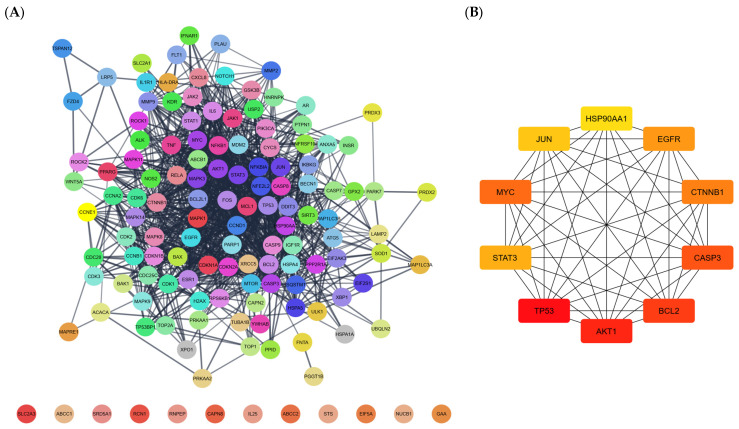
(**A**) The protein–protein interaction (PPI) network was constructed by submitting the list of the reported targets from Table 3 into the stringApp within the software Cytoscape; each coloured bubble represents a molecular target containing the protein gene name, and lines indicate connections between any given nodes; arranged according to yFiles Organic Layout. (**B**) Top 10 hubs after topological analysis using the CytoHubba plugin; the redness of the colour represents degree centrality.

Manually screening the 69 studies from Table 2 yielded 136 different protein and pathway targets, ranging from receptors to signal transductors and even structural proteins such as tubulin (Table 3). Eight targets were reported exclusively in silico, 100 in vitro, and 28 via both methods. Nearly all proteins were found within the STRING database, except for the vitamin d receptor and vascular endothelial growth factor a. Network construction was done within Cytoscape (Figure 2A), and subsequent topological analysis revealed p53 as the node with the highest degree centrality (hubs; Figure 2B). Notably, there was significant overlap between the top 10 hubs and the nodes with the highest betweenness centrality, indicating bottlenecks (Supplementary Figure S1). The raw data of the Cytoscape session is available through the Supplementary File S4 “sd4 134_GTs_targets.cys”.

**Figure 3:**
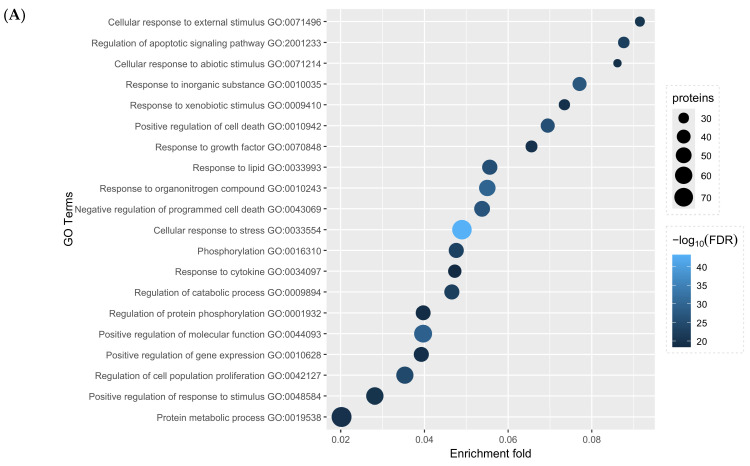

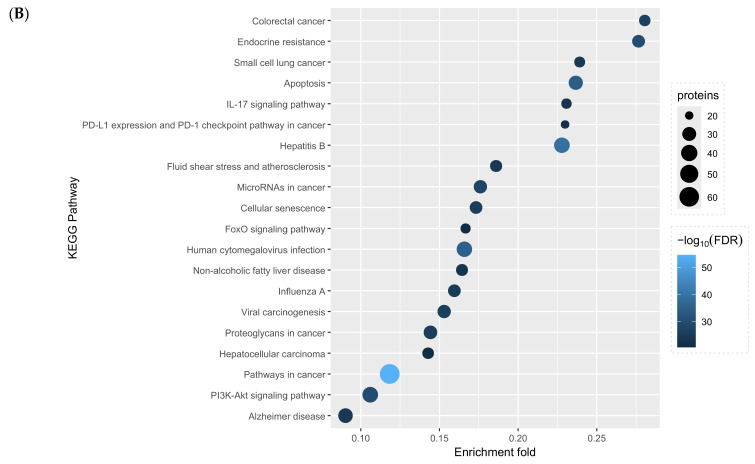
The bubble chart shows the (**A**) top 20 most enriched Gene Ontology Biological Processes (GO:BP) and (**B**) Kyoto Encyclopaedia of Genes and Genomes (KEGG) pathways. Bubble size correlates to the number of proteins from the network found within the enriched term, colour indicates statistical significance, and the *x*-axis indicates the enrichment fold calculated as the number of proteins found divided by the number of total proteins registered in the term. Analysed with stringApp through Cytoscape and plotted with RStudio’s ggplot2 package.

Gene Ontology–Biological Process (GO:BP) and Kyoto Encyclopaedia of Genes and Genomes (KEGG) pathway enrichment analysis was performed on the GTs network from Figure 2A. Bubble charts were constructed to highlight the top 20 most significant terms for GO or KEGG enrichment (Figure 3). A trend is observed in which the overall GO processes relate to responses against foreign agents and stimuli, responses to stress, and regulation of death. As for KEGG, expectedly, pathways enriched are linked to cancerous processes, but many are also associated with immune signalling. Notably, one of the most significantly enriched pathways was related to the regulation of PD-L1 and PD-1, two critical and sought-after targets in oncotherapy, considered major immune checkpoint modulators [146].

The present review is predominantly exploratory in nature; as such, in silico evidence was considered to build a theoretical framework that could link anticancer GT effects with the ICD phenomenon. However, to assess the robustness of a hypothetical bridge between GTs and ICD, the same enrichment analyses were performed again, considering only the 127 GTs’ molecular targets with physical in vitro evidence mentioned in Table 3. Without the in silico evidence, enrichment of the now “in vitro-exclusive” network reveals significant overlap between the most enriched terms, with 18 out of the top 20 GO:BP terms from Figure 3A still present. However, the two terms absent in this newly calculated top 20 GO:BP list, GO:0007166 (cell surface receptor signalling pathway) and GO:1901214 (regulation of neuron death), were still within the top 25 most significantly enriched GO:BP terms, with false discovery rate (FDR) values of 1.69^−21^ and 2.67^−18^, respectively.

Regarding KEGG enrichment, terms from Figure 3B were also present in the “in vitro-exclusive” node analysis, with FDR values indicating high statistical significance. For example, the “PD-L1 expression and PD-1 checkpoint pathway in cancer” KEGG term was originally enriched with an FDR value of 1.27^−22^ within the whole network, whereas in the 127-node in vitro network, it was enriched with an FDR value of 4.11^−20^. This term was also present within the top 25 most enriched KEGG terms of such “in vitro-exclusive” network. This Cytoscape session and the enrichment table raw data are available in the Supplementary File S7 “sd7 127_in_vitro_network.zip”.

### 3.4. Intersection of GT Targets and ICD-Related Proteins

As mentioned in the introduction, this review aims to construct a theoretical framework linking the anticancer effects of *Ganoderma* triterpenoids (GTs) and a potential induction of immunogenic cell death (ICD). Thus, a list of proteins involved in ICD was constructed as described in the Materials and Methods section and can be consulted in the Supplementary File S3 “sd3 icd_-_retrieval.ods”.

**Figure 4:**
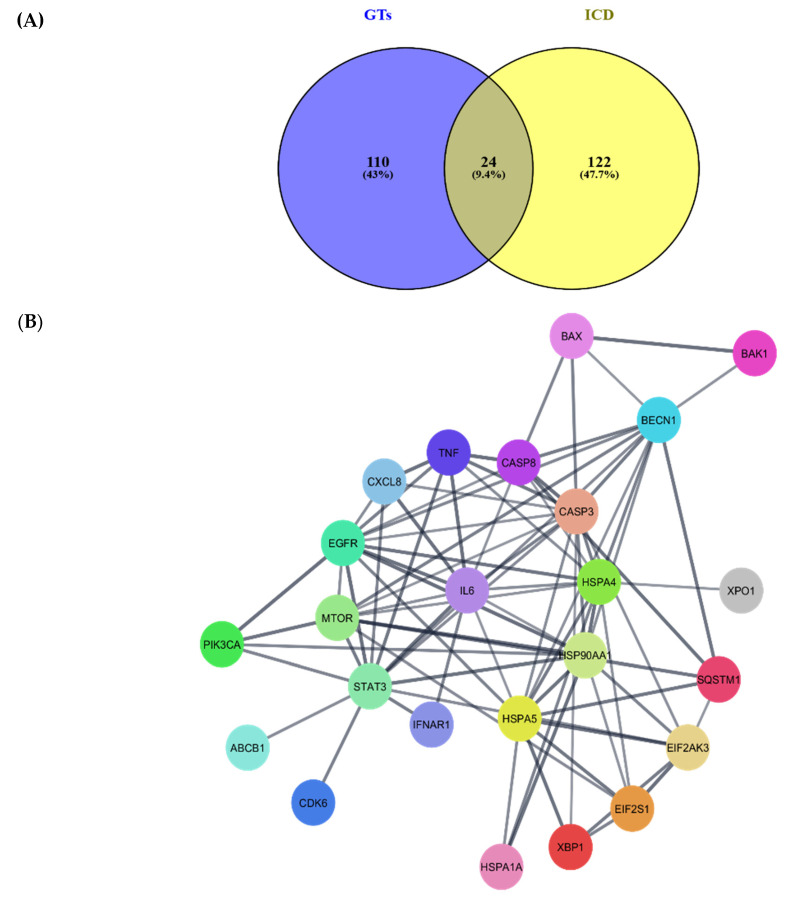

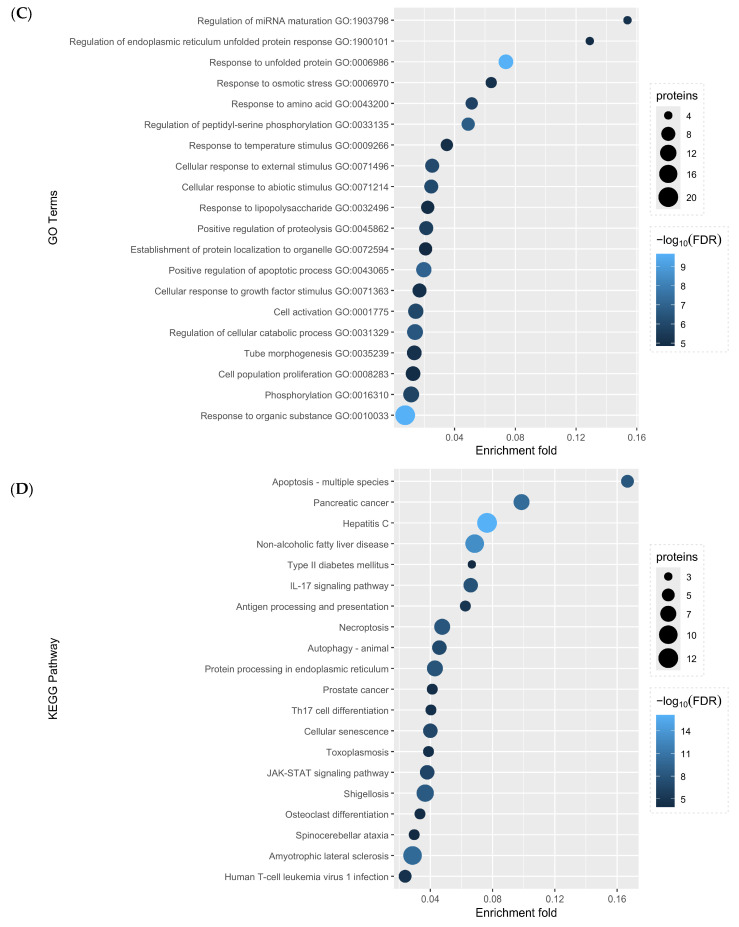
(**A**) Venn diagram showing intersecting molecular candidates between *Ganoderma* triterpenoid affected targets (GTs) in blue and participants of immunogenic cell death (ICD) in yellow, with an overlap of 24 putative targets. (**B**) Cytoscape protein–protein interaction (PPI) network of the 24 GT-ICD intersecting targets. (**C**) Enriched GO:BP and (**D**) KEGG terms calculated from the 24 intersecting targets network.

To establish a putative GTs-ICD connection, 24 intersecting targets between the two groups were identified in a Venn diagram (Figure 4A), and a new PPI network was constructed (Figure 4B). This network was considered the common ground between GTs and ICD. It is noteworthy that members of the heat shock proteins HSP70 (HSPA1A, HSPA4, HSPA5) and HSP90 (HSP90AA1), which are key DAMPs for the induction of ICD [82,89], were also present in the GT target protein group. Subsequent enrichment analysis on this network revealed the top 20 most significant GO:BP terms and KEGG pathways (Figure 4C). Overall, the anticancer effects of GTs and ICD induction appear to coincide in the context of stress and the unfolded protein response, death regulation, and immune processes.

## 4. Discussion

### 4.1. General (Non-Protein) Effects Observed After Treating Cancer Cells with GTs

Table 2 reveals an evident trend: a single *Ganoderma* triterpenoid (GT) isoform can elicit multiple cellular effects and/or affect different molecular targets across various human cancer cell lines. The inverse is also true: the same cellular changes and affected molecular targets in each cancer cell type were observed with multiple GT isoforms. This could be related to the fact that, despite structural variation, the common denominator between them is a lanostane core precursor. This shared chemical origin could be conferring GTs with similar effects against cancer. For example, cell cycle arrest was a phenomenon observed with ganoderic acid (GA)-A, GA-D, GA-DM, GA-Me, GA-Mf, GA-S, GA-T, other non-GA GTs, and triterpenoid mixtures (Table 2). Other effects shared amongst GTs were DNA damage, increased oxidative stress and/or reactive oxygen species (ROS), reduction in mitochondrial membrane potential (↓ΔΨm), and autophagy.

Cell cycle arrest has been associated with DAMP emission, particularly with the natural product-derived medication paclitaxel [147]. The primary mechanism of radiotherapy is DNA damage, a process also linked to DAMP release and the induction of cancer cell ICD [148,149]. Similarly, anthracyclines, which are chemotherapeutics derived from bacterial natural products, directly damage DNA and induce ICD via DAMP emission [150,151]. Furthermore, the mechanisms for releasing high-mobility group box 1 (HMGB1), a key DAMP, are directly tied to DNA damage signalling [152]. Another phenomenon that can potentially imbue cell death with immunogenic properties is the increase in ROS, regardless of the cell death modality [153,154], because oxidative damage to the endoplasmic reticulum (ER) can lead to outer membrane translocation of calreticulin (CALR), another quintessential DAMP for ICD [155]. Knowing that GTs can induce cell cycle arrest, DNA damage, and ROS increase, and considering that all of these phenomena have been reported elsewhere to induce DAMP emission, could a connection be drawn between *Ganoderma* triterpenoids and ICD? We hope to continue discussing and constructing an answer below.

Reducing ΔΨm implies a compromised external mitochondrial membrane, a phenomenon observed after pore formation during cellular stress and aptly named mitochondrial outer membrane permeabilization (MOMP) [156]. If MOMP-pore formation reaches a critical size, the inner mitochondrial membrane herniates and releases the organelle’s contents into the cytosol, including mitochondrial DNA (mtDNA), thereby acting as DAMPs [157]. Because mitochondria are descendants of bacteria, their DAMPs also display pathogen-associated molecular pattern (PAMP) characteristics, with a tendency to elicit Type I interferon (IFN) responses [158]. As observed in Table 2 and Table 3, GTs tend to reduce the ΔΨm of cancer cells concomitantly with an increase in the bcl-2-like protein 4 (BAX) and Bcl-2 homologous antagonist/killer (BAK) proteins, which are key molecular drivers of MOMP [156,157]. Thus, there is a possibility that when GTs trigger MOMP, this effect could be followed by the emission of mtDNA as a DAMP. However, to go beyond mere conjecture, experimental confirmation is required, perhaps by treating cancer cells with GTs and measuring Type I IFN signalling after inducing such mitochondrial alterations.

### 4.2. Reported GT Protein Targets

As mentioned previously, any given specific isoform of *Ganoderma* triterpenoid (GT) is observed to affect diverse molecular targets in cancer cells, and different GT isoforms frequently converge in observed molecular outcomes (Table 2 and Table 3). This means that GTs as a whole exhibit polypharmacological action. Such pleiotropy and redundancy of GTs is reminiscent of the molecular behaviour also observed with cytokines [159]. While this duality may complicate the determination of a concise mechanism of action, it offers the potential benefit of curbing cancer cell therapeutic resistance. As such, polypharmacological action has become an increasingly coveted property sought out for future treatments [160,161]. Regarding ICD, combinations of agents with narrow mechanisms tend to be more successful at killing cancer cells in an immunogenic fashion [162]; therefore, an argument could be made that the multi-pharmacological action of GTs could increase the likelihood of exerting ICD in cancer cells.

Unfortunately, the wider the pharmacodynamic nature of a given therapeutic agent, the higher the possibility of engaging off-targets resulting in adverse effects. For this reason, “antitarget” databases are currently being designed to preliminarily screen binding of therapeutic prospects, thereby increasing rigor when transitioning from preclinical to clinical research [161]. Therefore, it is crucial to investigate whether GTs can engage with or not with such undesired molecular targets.

In the context of ICD potential from GTs, the best “smoking gun” so far could be that certain DAMPs and DAMP-emission-associated proteins were explicitly altered by GT treatment in cancer cells (Table 2 and Table 3). In a study, ganoderic acid (GA)-D affected several prostate cancer proteins in vitro that were later subjected to protein interaction network analysis in silico that revealed heat shock protein 90 kDa alpha member A1 (HSP90AA1), heat shock cognate 71 kDa protein (HSPA8), and exportin 1 (XPO1) as putative mechanistic components of GA-D’s anticancer effects [138]. This suggests a potential connection between GA-D and ICD, because XPO1 is required for the emission of high-mobility group box 1 (HMGB1) protein [163] and the translocation of calreticulin (CALR) to the outer cell membrane [164]; thus, XPO1 allows both DAMPs to become available for binding with pattern recognition receptors (PRRs) located on dendritic cells (DCs) mentioned in Table 1. Although HSPA8 is not typically mentioned as an ICD-associated DAMP, it can also activate Toll-like receptor 4 [165]. However, GA-D’s association with these HSPs and XPO1 is only supported in silico, for which further in vitro validation is required.

Additionally, two out of the retrieved 69 studies reported anticancer GT effects via induction of endoplasmic reticulum stress (ERS). In one such study, GA-DM treatment on prostate cancer cells increased the expression of heat shock 70 kDa proteins (HSP70), C/EBP homologous protein (CHOP, also known as DDIT3), and calpain. In the same study, pre-treated cancer cells with GA-DM significantly increased the activation of cocultured T cells [10]. Similarly, hepatocarcinoma cells treated with an extract containing ganoderic, ganoderenic, and lucidenic acids were reported to have higher levels of PKR-like endoplasmic reticulum kinase (PERK) expression, eukaryotic translation initiation factor 2α phosphorylation (p-eIF2α), and CHOP protein [105]. This could be considered as further evidence connecting GTs and ICD. Other authors have demonstrated that p-eIF2α and CHOP are related to the release of CALR and HMGB1 [155,166]. But beyond that, p-eIF2α per se is considered a hallmark of ICD [167]. The mechanistic explanation is that PERK phosphorylates eIF2α, and p-eIF2α then allows activating transcription factor 4 (ATF4) to increase CHOP transcription. CHOP acts as a transcription factor for proteins involved in ERS-related cell death [168,169]. Calpains also enhance antigen cross-presentation towards CD8^+^ T cells [170] and potentially participate in heat shock-induced ICD [171].

However, heat shock proteins (HSPs) are DAMPs only when translocated and exposed to the outer membrane of dying cancer cells, because this is how such molecules become engageable by PRRs on DCs (Table 1). Therefore, further studies are needed to rigorously confirm whether the increase in HSP70 observed after treating cancer cells with GA-DM goes beyond mere intracellular accumulation.

### 4.3. GT Enrichment Analysis Suggests an Immunological Component

As seen in Figure 3A, functional enrichment of the affected targets listed in Table 3 reveals that processes most associated with GT treatment are centred around responding to exogenous stress and the regulation of death. Turning our attention to the KEGG pathways highlighted in Figure 3B, the enrichment of immunological pathways is particularly notable. Interestingly, the enrichment related to PD-L1/PD-1 (KEGG hsa05235) suggests a potential mechanism by which GTs affect the expression of this immune checkpoint. Such enrichment maintained a very high statistical significance even when excluding in silico evidence (Supplementary Data within Supplementary File S7 “sd7 127_in_vitro_network.zip”). Programmed death-ligand 1 (PD-L1, a.k.a. CD274), as its name suggests, is the physiological agonist of programmed cell death protein 1 (PD-1, a.k.a. CD279). PD-1 is a receptor primarily observed on lymphocytes, and its activation by PD-L1 is crucial for ending immune reactions and promoting tolerance. Unfortunately, cancer abuses this mechanism to escape the immune system and perpetuate, making PD-L1/PD-1 mainstay targets for immunotherapies [172].

As observed in Table 2, GTs downregulate numerous PD-L1/PD-1 (KEGG hsa05235) pathways, including AP-1 (c-jun/c-fos), ERK, JAK/STAT, NF-κB, and PI3K/Akt/mTOR pathway-related proteins. Therefore, it seems that GTs not only cause cancer cell death but might also downregulate crucial immunosuppressive factors, such as PD-L1. Confirming such a scenario with experimental evidence would grant immune checkpoint inhibitor (ICI) status to *Ganoderma* terpenoids. Combining ICD inducers and ICIs is a tremendously contested topic. Both great success and significant failure have been observed with the ICD-ICI combination, indicating that much remains to be explored. Current efforts focus on the timing and dosing of each group of agents during combinatorial regimes [173,174]. However, no data currently exist of any agent capable of acting simultaneously as an ICI and ICD inducer; thus, experimental validation could pave the way for GTs to become the first compounds exhibiting this dual mechanistic nature.

### 4.4. GTs and ICD Converge Mainly on Cancer Cell Stress, Perforation, and Impaired Proteostasis

As mentioned earlier, BAX and BAK were amongst the most upregulated proteins following treatment of cancer cells with GTs (Table 2). This protein duo is also involved in ICD (Figure 4B), as BAX/BAK activation is essential for CALR exposure during cancer death by chemical [155] and physical means [42]. Mechanistically, stress-induced cell death activates BAX/BAK, which then perforate the mitochondrial and ER membranes. Pore formation leads to calcium efflux into the cytosol [175,176], and this ER depletion of Ca^++^ induces the translocation and external membrane exposure of CALR [177]. Additionally, MOMP caused by BAX/BAK can lead to the release of mtDNA and mt-dsRNA into the cytosol, activating the cGAS/STING and RIG-I/MAVS pathways within cancer cells, which elicits the secretion of type I IFN cytokines for ICD induction [158]. Furthermore, mitochondria, their content, and pre-activated cGAS/STING machinery are susceptible to intercellular transfer, leading to amplification of DAMP signalling and immune activation [178,179]. Thus, such BAX/BAK perforation might link GTs with ICD potential. This could be confirmed by measuring whether GT-induced BAX/BAK activation in cancer cells is followed by extracellular secretion of mitochondrial DAMPs.

Results from Figure 4B confirm caspases as another coinciding aspect between GTs and ICD. These proteases are essential in modulating programmed cell death, with their role in ICD being context-dependent. Caspases were found to be required for doxorubicin’s ICD effects [150] and for the immunogenic amplification of cancer demise by CD8^+^ T cells [180]. The primary ICD-related mechanism of caspases is the proteolytic cleavage of gasdermins (GSDMs). This can change programmed death modalities with a tendency towards pyroptosis [181,182]. Such is the caspase-3 cleavage of GSDME, which releases the latter’s N-terminal domain. Cleaved GSDME then perforates cellular and mitochondrial membranes, culminating in cell rupture with DAMP release [183,184]. Cleavage of GSDMC [185] and GSDMD [186] by caspase-8 results in similar outcomes. Finally, caspase-8 cleavage of Bap31 can activate BAX/BAK, leading to anterograde Golgi transport and exposure of CALR [155,187]. Because caspases were amongst the most reported activated proteins by GTs, there is a possibility of inducing ICD with such triterpenoids through caspase-driven activation of GSDMs.

Enrichment analysis was performed to gain insight into the relationships interconnecting these 24 GT-ICD intersecting proteins. Figure 4C shows a notable enriched theme: unfolded protein response (UPR). When proteostasis is altered following ER stress (ERS), cells activate the UPR, a pathway that increases expression of folding chaperones, halts synthesis of other proteins, and increases misfolded protein degradation [188]. Physiologically, this helps normal cells, but cancer can harness the same UPR process to heighten oncogenic programming. However, intense and/or irreparable ERS can redirect cancerous UPR into a potent ICD mechanism through the main arms of the UPR driven by PERK phosphorylation of eIF2α [189]. Such an event is also considered pathognomonic of ICD [166]. As previously discussed, two studies reported the activation of ERS and UPR after treating cancer cells with either the specific triterpenoid GA-DM [10] or a GT-enriched extract [105]. Considering the nature of this latter mixture, more studies are required to elucidate which, if any, of the mixture triterpenoids was responsible for such ERS-UPR anticancer effect.

### 4.5. Counterarguments Against the Possibility of a GT-ICD Connection

Unfortunately, not all evidence indicates a valuable connection. First and foremost, no experiment exists linking GTs and ICD. Secondly, the same anticancer effects observed with GTs could well interfere with the ICD process. As observed in Table 2, GTs downregulate and inactivate proteins involved with PI3K/Akt/mTOR, NF-kB, and TNF signalling. While these pathways are exploited by cancer for its growth, transformation, invasiveness, and PD-L1 expression, the same pathways are also crucial for the proper maturation, differentiation, recruitment, and survival of immune cells [190]. Additionally, GTs promote autophagy and apoptosis in cancer cells (Table 2). These cell death modalities are vital homeostatic processes that continuously happen in a housekeeping manner. Unsurprisingly, they mainly induce immunosilent cell death on their own. This is because apoptotic caspases and autophagic pathways aim to degrade proteins, including antigens, DAMPs, and the machinery responsible for DAMP emission [191]. Furthermore, successful ICD has been reported after cell death combined with caspase inhibition [158] or autophagy blockage [192]. Additionally, GTs have been reported to be antioxidant and anti-inflammatory [3]. Thus, it could be argued that GTs might limit immune activation required for ICD.

However, other research counters this notion, suggesting that autophagy is a crucial step in ATP emission as a DAMP [191,193] and that caspase activation is required in certain instances of ICD, such as with doxorubicin [150]. Moreover, a recent study with multiple cancer cell lines confirmed that HMGB1 release for ICD requires caspase activation, illustrating the striking ambivalence of cell death-related processes [194]. Furthermore, current knowledge on the subject proves that regulated cell death modalities are not isolated pathways, but rather intricately intertwined [195].

Another point of contention is the fact that p53 was deemed the most significant molecular node within the GTs targets network (Figure 2B). p53 mutations confer cancer resistance to death, and such protein aberration is amongst the most common somatic mutations [162]. However, ICD as a phenomenon appears to be p53-agnostic, because even when a cancer cell can resist certain regulated forms of death, such as those that would be p53-dependent, this does not mean that the same cancer cell would be impervious to alternate pathways activated after being exposed to lethal stress [162]. As observed in Table 2, GTs exert anticancer effects on p53-mutated cells such as DU-145 [196] and MDA-MB-231 [197], as well as on p53-null cell lines such as PC-3 [196] and HL-60 [198]. Therefore, it appears that p53 status does not affect resistance to the anticancer effects of GTs.

As such, consensus cautions against labelling particular regulated cell death modalities or associated phenomena as strictly immunogenic or tolerogenic, calling for nuance and appropriate experimental validation of DAMP emission and immune activation on a case-by-case basis [162]. Therefore, it is also technically possible to argue that GTs might have the potential to hamper DAMP emission the requirement for ICD, mainly through non-immunogenic forms of regulated cell death, interference with DAMP-releasing machinery, or through anti-inflammatory effects.

### 4.6. In Vivo Evidence for a Potential GT-ICD Relationship

The inclusion criteria of the articles retrieved for the present review limit cancer cells to those of *Homo sapiens* origin. As is often the case with bioactive natural products, evidence for the anticancer effects of *Ganoderma* triterpenoids (GTs) in humans is practically non-existent. The closest available information is from suboptimal clinical trials using crude preparations and extracts without specification of triterpenoid content. It is worth mentioning that amongst the *Ganoderma*-derived compounds, polysaccharides have been clinically assessed [199]. Fortunately, some clinical evidence supports the notion that *Ganoderma* preparations have neither severe nor moderate adverse effects in cancer patients and may even provide significant symptomatic relief [200,201]. Unfortunately, no clinical literature exists focusing on triterpenoids from *Ganoderma* in the context of cancer.

However, if we temporarily expand our criteria to consider other animal models, a broader picture emerges, tipping the scales in favour of a positive pro-immunogenic anticancer potential of GTs. Firstly, GTs undoubtedly have anticancer effects in vivo. This has been proven in animal models of various cancer types such as breast [17,202], colorectal [203], liver [204,205], lung [14,206,207,208], lymphoma [24], melanoma [23], ovarian [25], and sarcoma [209]. Thus, it can at least be argued that the GTs’ anticancer effects do not interfere with the immune function of a living host.

Perhaps the most impressive aspect is the in vivo evidence demonstrating how GTs exert anticancer effects by enhancing antitumoral immunity. A study reported increased function of NK and CD8^+^ T cells, as well as increased interleukin-6 (IL-6) production, following the in vivo administration of an extract containing at least 34.45% GTs. Additionally, the GTs were found to synergize with cyclophosphamide, alleviating its side effects and abolishing metastasis [26]. Another study utilized GA-A and reported direct antitumoral and antimetastatic impact, a reduction in myeloid-derived suppressor cells, and enhancement of CD8^+^ T cell function [24]. GA-DM exhibited similar effects by promoting tumoral apoptosis and autophagy, augmenting MHC-II antigen presentation, and increasing tumoral infiltration, thereby enhancing antigen recognition by CD4^+^ T cells in vivo [23]. Yet another triterpenoid, GA-ME, increased in vivo NK cell activity and the antitumoral cytokines IL-2 and IFN-γ, with the effect attributed to increased NF-κB signalling [27]. Finally, GA-T reprogrammed the tumoral microenvironment and enhanced immune function, with additional synergistic effects when combined with paclitaxel or the anti-PD-L1 antibody atezolizumab [25].

Collectively, this in vivo evidence regarding the anticancer effects of GTs supports the idea that, at the very least, GTs are well-tolerated molecules with positive anticancer immune effects. To understand whether these effects are solely a result of boosting the physiology of immune cells or if there is a bona fide ICD component to it, a direct assessment of the machinery involved in such processes and confirmation of immune memory generation will be required.

### 4.7. Limitations and Future Prospects

Variables inherent to the databases consulted, such as the English language, indexing, and publication bias, may prevent the inclusion of relevant literature for a more comprehensive analysis and/or skew the overall direction of the data. Secondly, the research analysed is entirely preclinical; in silico calculations and in vitro culture of cancer cell lines hardly extrapolate into a real clinical setting. We must never forget that any cancerous process is entirely beholden to every variable within a host organism. Thirdly, experimental design limitations include the limited implementation of non-cancerous cells, the lack of positive controls to benchmark the GTs against, the use of non-standardized extracts, the lack of kinetic analysis of GTs and/or targets reported, and heterogeneity in the assays and cell lines, amongst others. Finally, since humans conducted the overall study and discussion of the present review, there is always the risk of interpretation bias.

Another limitation is the absence of a PROSPERO protocol registration for the present review. A preliminary review of the available evidence revealed no human in vivo evidence with GTs, which is juxtaposed with the hypothetical nature of a preclinically proposed GT-ICD link. Thus, it was deemed that our review would not meet the direct clinical weight often expected from more clinically impactful systematic reviews.

In the future, specific strategies can be implemented to overcome these limitations and start research regarding GTs and ICD. First and foremost, experiments quantifying proper emission and spatiotemporal distribution of DAMPs following GTs treatment of cancer cells are warranted. Should the pharmacology of GTs be the focus, ^14^C and/or ^3^H-labelled triterpenoids could be used to track kinetics within cancer cells through electron microscope autoradiography; alternatively, immunofluorescence could achieve the same objective. Pharmacodynamic research could be more adequately guided after the subcellular localization of GTs is well studied. Regarding disease modelling, human cell organoids could serve as an adequate middle ground between 2D in vitro cultures and whole-organism in vivo models. Finally, cancer organoids treated with GTs co-cultured with immune cells would be ideal for measuring the later stages of ICD, when antitumoral immune engagement is expected, thus allowing researchers to assess the effective potential of ICD.

## 5. Conclusions

Structural differences exist among *Ganoderma* triterpenoids (GTs). Still, their lanostane core confers them common physicochemical properties, enabling them to infiltrate cellular compartments and alter the cancer machinery in convergent, polypharmacological ways. The mainstays of anticancer GTs’ action seem to be mitochondrial dysfunction, stress, slowing cellular function, and even halting it altogether. An undeniable overlap exists between the proteins affected in cancer cells treated with GTs and those involved in ICD, particularly in the case of the triterpenoid GA-DM. Looking only at the proteins that GTs altered within human cancer cells could cast some doubt on a potential connection to ICD, due to the downregulation of key pathways in cancer that are also necessary within immune cells for antitumoral immunity and ICD. However, the in vivo non-human evidence starkly contrasts this assumption, as multiple antitumoral effects of GTs are seen concomitantly with potentiation of immune function, and incredible synergism with chemotherapy. Furthermore, enrichment analysis confirms a potential immune checkpoint inhibition effect of GTs. Finally, these compounds have not shown any significant toxicity even when administered to chemotherapy patients. Therefore, GTs are anticancer compounds with immune-boosting functions and a tangible potential to induce ICD. With direct experimental analysis, this connection could be confirmed.

## Figures and Tables

**Table 1:** List of damage-associated molecular patterns (DAMPs), their respective pattern recognition receptors (PRRs), and their effects on dendritic cell (DC) function.

DAMP	PRR Activated	Effect on DCs	Reference
ATP	P2X7	NLRP3 inflammasome activation Migration Cross-dressing presentation	[34,35,36]
	P2Y11	Activation and maturation Modulation of inflammation Thrombospondin-1 secretion	[37,38,39]
CALR	CD91 (LRP1)	MHC-I and -II antigen presentation Activation and maturation Pro-phagocytic	[40,41,42]
	SREC-1 (SCARF1, SR-F1)	Apoptotic cell clearance C1q binding	[43,44]
HMGB1	CD24	Discrimination between DAMP and PAMP contexts Captures and presents HMGB1 to CD8+ cells’ RAGE receptor	[45,46]
	CXCR4	Migration	[47]
	RAGE	Regulation of homing receptors CCR7 and CXCR4 Chemotaxis Maturation Blocking of apoptotic tolerance	[48,49,50,51]
	TLR2	Activation Antigen presentation Maturation	[52,53]
	TLR4	Licensing Antigen presentation Migration	[54,55,56]
	TLR9	Increases CpG-DNA sensing Activation	[57]
HSP70	CD40	Antigen uptake	[58,59,60]
	CD91 (LRP1)	Antigen internalization MHC-I and MHC-II presentation Memory T cell generation	[40,61,62,63]
	LOX-1	Antigen uptake MCH-I and MHC-II presentation	[64,65,66]
	SREC-1 (SCARF1, SR-F1)	Antigen internalization Cross-presentation	[67,68]
	TLR2	Activation Maturation MyD88 signalling Increased expression of SREC-1	[67,69]
	TLR4	Activation Maturation Chemotaxis MyD88 signalling T_H_1 response	[55,69,70,71]
HSP90	CD91 (LRP1)	Antigen internalization Immunosurveillance MHC-I and MHC-II presentation	[40,66,72,73]
	LOX-1	Cross-priming MHC-I and MHC-II presentation Antigen uptake	[65,66,74]
	SREC-1 (SCARF1, SR-F1)	Cross-priming MHC-I and MHC-II presentation Antigen uptake	[65,74,75]
	TLR2 and TLR4	Activation Innate and adaptive immune response amplification	[76,77]
	TLR9	T_H_17 T cell polarization MHC upregulation Increases CpG-DNA sensing	[78,79]

**Table 2:** List of studies included in the systematic review.

Study	*Ganoderma* Species	Compounds	Essay Type	Cancer Cell Lines	Affected Targets	Other Observed Effects	Safer for Non-Cancerous Cells
[9]	None	GA-A, GA-DM	in vitro	CH157MN, IOMM-Lee	↑ Bax, ↓ Bcl-XL, ↓ Mcl-1, ↓ c-myc, ↑ caspase-3, ↓ Cyclin D1, ↑ Frizzled-4 (CD344), ↑ GSK3β, ↑ LRP5, ↓ Akt, ↓ TSPAN12, ↓ VEGF, ↓ Wnt/β-catenin pathway	DNA fragmentation, apoptosis	Yes, healthy neurons
[95]	*G. lucidum*	GTs	in vitro	DU-145	↓ MMP2/9	N/R	N/R
[96]	*G. colossum*	Lucidenic Acid mixture	in vitro	HepG2	↓ AP-1, ↓ ERK1/2, ↓ MMP9, ↓ NF-κB, ↓ Akt	N/R	N/R
[97]	*G. lucidum*	GA-T	in vitro	95-D	↓ MMP2/9, ↓ NF-κB	N/R	Yes, HEK293 and LO2
[98]	*G. lucidum*	GA-A	in vitro, in silico	IMR-32	docks and ↓ Notch-1 mRNA	N/R	N/R
[99]	*G. lucidum*	GA-Mk	in vitro	HeLa	↑ caspase-3, ↑ caspase-9	↑ ROS, ↓ ΔΨm, apoptosis	N/R
[20]	*G. gibbosum*	Gibbosic Acid H	in vitro	MDA-MB-231, SNU638, SK-Hep-1, A549, H1299	↑ ACC (Acetyl-CoA carboxylase), ↑ AMPK, ↑ Bax, ↓ Bcl-2, ↑ Beclin 1, ↑ caspase-3, ↑ caspase-8, ↓ CDK22, ↓ Cyclin D1, ↓ Cyclin E1, ↑ LC3B, ↓ Sequestosome-1, ↑ p21 (CDKN1A), ↑ p53, ↑ ULK1	Autophagy, G0/G1 cell cycle arrest, apoptosis	N/R
[100]	*G. lucidum*	GA-T	in vitro	HeLa	↑ caspase-3, ↑ caspase-9	G1 cell cycle arrest, ↓ DNA synthesis, ↓ ΔΨm, apoptosis	Yes, for “non tumour” MCF
[101]	*G. lucidum*	12α-MeO-Ganodermanondiol	in vitro, in silico	MDA-MB-231, HepG2	docks TNF	N/R	N/R
[5]	None	GA-A, GA-H	in silico	n/a	docks NEMO (IKK-γ)		
[16]	None	GA-A, PROTACs C1–C10 and V1–V10	in vitro, in silico	SJSA-1, MCF7, MDA-MB-231, HepG2	↑ Bax, ↓ Bcl-2, ↓ Cyclin D1, ↑ p21 (CDKN1A), ↑ p53; docks and ↓ MDM2 protein	N/R	N/R
[19]	None	GA-A	in vitro, in silico	A549	docking and thermal shifting of ↓ GLUT1/3	N/R	N/R
[22]	None	GA-D	in vitro	HT29, SW620	↓ Peptidylprolyl isomerase D (cyclophilin D), ↑ SIRT3	Inhibits the Warburg effect	N/R
[102]	*G. lucidum*	GA-Me	in vitro	HCT8, HCT116	↓ ABCB1 (MDR1), ↑ Bax, ↓ Bcl-2, ↑ caspase-3, ↑ caspase-9, cytochrome-c release, ↓ MRP1/2 (ABCC1/2), ↑ p53	Reversed multidrug resistance, ↓ ΔΨm	N/R
[103]	*G. lucidum*	GA-F, GA-K, GA-B, GA-D and GA-AM1	in vitro	HeLa	↑ 14-3-3, ↑ TPM4-ALK fusion oncoprotein type 2, ↓ chain A of DJ-1, ↓ eIF5A, ↓ HNRNPK (Heterogeneous nuclear ribonucleoprotein K), ↓ IL-17E, ↓ Nucleobindin 1, ↓ Peroxiredoxin 2, ↓ PPP2R1A, ↓ Reticulocalbin-1, ↓ SOD1, ↓ Ubiquilin-2	N/R	N/R
[18]	None	Various GTs in silico, GA-DM in vitro	in vitro, in silico	MCF7, MDA-MB-231	docks STAT1 and AKT1; docks and ↓ β-catenin, ↓ PIK3CA, ↑ EGFR mRNA; ↓ PIK3CA protein	N/R	N/R
[10]	None	GA-DM	in vitro	LNCaP, PC-3	↑ Atg5, ↑ Bax, ↓ Bcl-2, ↑ Beclin 1, ↑ calpain 2, ↑ calpain 8, ↑ caspase-3, ↑ CHOP (DDIT3), ↑ GRP78 (BiP), ↑ HLA-DR, ↑ HSP70, ↑ LC3-II	Autophagy, G2 cell cycle arrest, ER stress, DNA fragmentation; ↓ ΔΨm, apoptosis; coculture with T cells: ↑ IFNγ	N/R
[104]	*G. lucidum*	GA-A	in vitro	PC-3	docks SH2 domain of STAT3	↓ total mRNA	N/R
[105]	*G. sinense*	Sporoderm extract contains GA-A, GA-B, GA-D, LA-D, ganolucidate F	in vitro	HepG2	↓ CDC25C, ↓ CDK1, ↑ CHOP (DDIT3), ↓ Cyclin A2 (CCNA2), ↑ eIF2α (EIF2S1), ↑ GRP78 (BiP), ↑ PERK (EIF2AK3), ↑ XBP1s/XBP1u ratio	G2/M cell cycle arrest, dilation of the ER, ER stress and UPR activation	N/R
[106]	*G. lucidum*	GA-A, amide derivative	in vitro, in silico	SJSA-1, MCF7, HepG2	↑ Bax, ↓ Bcl-2, ↑ p53; binds to ↓ MDM2 and interaction with p53	N/R	Moderately with HK-2
[107]	None	GA-A	in vitro	MDA-MB-231	↑ Bax, ↑ Bak, ↓ Bcl-XL, ↓ Mcl-1, ↓ Cyclin D1, cytochrome-c release, ↓ JAK2, ↓ STAT3, ↑ p21 (CDKN1A), ↑ p27 (CDKN1B)	G0-G1 cell cycle arrest, ↑ ROS	N/R
[108]	*G. lucidum*	GA-A	in vitro	U251	↑ Bax, ↓ Bcl-2, ↑ Beclin 1, ↑ caspase-3, ↓ Cyclin D1, ↑ LC3-II, ↓ Sequestosome-1, ↓ Akt, ↓ mTOR, ↓ RPS6KB1	Autophagy	N/R
[109]	None	GA-A	in vitro	SMMC7721, HepG2	↑ caspase-3, ↓ Cyclin D1, ↑ p21 (CDKN1A)	G0/G1 cell cycle arrest	N/R
[15]	None	GA-A	in vitro	A549	↓ Beclin 1, ↓ LC3II/LC3I, ↑ Sequestosome-1	Anti-autophagy, reversal of cisplatin resistance	N/R
[110]	None	GA-A	in vitro	HOS, MG63	↓ STAT3, ↑ NF-κB1, ↑ p38 (MAPK14/11)	N/R	N/R
[111]	None	GA-A	in vitro, in silico	HeLa, A549	↑ caspase-3; docks and ↓ IL-1R1	↓ ΔΨm, apoptosis	N/R
[112]	None	GA-D	in vitro	EC9706, Eca109	↑ Beclin 1, ↑ caspase-3, ↑ caspase-7, ↑ Cyclin B12, cytochrome-c release, ↓ LAMP2, ↑ GFP-LC3, ↑ LC3B, ↑ LC3-II, ↑ Sequestosome-1, ↑ p53, ↑ PARP1, ↓ PI3K-AKT pathway	Autophagy, G2/M cell cycle arrest, ↑ ROS, destruction of autophagic flux, ↓ ΔΨm, apoptosis	N/R
[113]	None	GA-DM	in vitro	A549, NCI-H460	↑ Bax, ↓ Bcl-2, ↑ caspase-3, ↑ LC3B-I, ↑ LC3B-II, ↑ PARP1, ↓ PI3K-AKT pathway	Autophagy, apoptosis	N/R
[114]	*G. lucidum*	GA-DM	in vitro	MCF7	↓ c-myc, ↓ CDK22, ↓ CDK6, ↓ Cyclin D1, ↑ γH2AX, ↑ PARP1	G1 cell cycle arrest, DNA damage, ↓ ΔΨm	N/R
[115]	*G. lucidum*	GA-DM	in vitro	LNCaP, PC-3	↓ 5α-Reductase, ↓ AR (androgen receptor)	N/R	N/R
[116]	*G. lucidum*	GA-Me	in vitro	HCT116	↑ Bax, ↓ Bcl-2, ↑ caspase-3, cytochrome-c release	p53-G1 cell cycle arrest, ↓ ΔΨm, apoptosis	N/R
[117]	*G. lucidum*	GA-Me	in vitro	HeLa, HCT116, 95-D, H1299	↑ p53	p53-G1 cell cycle arrest	Yes, LO2 and HFL-1
[118]	*G. lucidum*	GA-Me	in vitro	95-D	↓ MMP2/9	N/R	N/R
[119]	*G. lucidum*	GA-Mf, GA-S	in vitro	HeLa, 95-D, HO-8910PM, SW1990	↑ Bax, ↓ Bcl-2, ↑ caspase-3, ↑ caspase-9, cytochrome-c release	G1 and S cell cycle arrest, ↓ ΔΨm, apoptosis	Yes, fibroblasts and LO2
[120]	None	GA-A	in vitro, in silico	MDA-MB-231, T-47D	docks and ↓ TNF, ↓ DR5 (TRAILR2)	N/R	N/R
[121]	*G. lucidum*	GA-T	in vitro	HeLa	↑ caspase-8, cytochrome-c release, ↑ γH2AX, ↑ TP53BP1	G1 cell cycle arrest, ↑ ROS, ↓ intracellular ATP, double-strand breaks, ↓ ΔΨm, necroptosis	N/R
[14]	*G. lucidum*	GA-T	in vitro	HCT116	↑ IκBα, ↓ iNOS, ↓ MMP2/9, ↓ p65 (RELA), ↓ TNF, ↓ uPA (PLAU)	N/R	N/R
[122]	None	GA-A, GA-C2, GA-C6	in vitro, in silico	HepG2	docks estrogen receptor, IGFR, insulin receptor, VEGR-1 and VEGR-2	N/R	N/R
[123]	None	GA-A	in vitro, in silico	H460	docks and ↓ Nrf2	↓ total mRNA	N/R
[124]	*G. amboinense*	GA-X	in vitro	HCT116, HL-60, Raiji (Burkitt’s lymphoma), HuH-7	↓ Bcl-XL, ↑ caspase-3, cytochrome-c release, ↑ ERK, ↑ JNK1/2, ↑ PARP1, ↓ DNA topoisomerase 1 and 2a	inhibition of DNA synthesis, cell shrinkage, degradation of chromosomal DNA, ↓ ΔΨm, apoptosis	N/R
[125]	None	GA-Me	in vitro	MDA-MB-231	↓ Bcl-2, ↓ c-myc, ↓ Cyclin D1, ↓ IL-6, ↓ IL-8, ↓ MMP9, ↓ NF-κB, ↓ VEGF	N/R	N/R
[126]	*G. lucidum*	GA-A, GA-H	in vitro	MDA-MB-231	↓ AP-1, ↓ CDK3, ↓ NF-κB, ↓ uPA (PLAU)	N/R	N/R
[127]	*G. amboinense*	Ganoderiol F (Gol-F)	in vitro	K562, HepG2, HuH-7	mild ERK phosphorylation, ↑ p16 (CDKN2A), ↓ p21 (CDKN1A), ↓ DNA topoisomerase 1 and 2	G1 cell cycle arrest, inhibition of DNA synthesis	Yes, MC5 and PBMC
[128]	*G. lucidum*	GA-B	in vitro, in silico	MCF7, HepG2	docks and ↓ ABCB1 (MDR1)	reversed multidrug resistance, efflux inhibition	N/R
[4]	*G. lucidum*, *G. tuberculosum*	GA-C2, GA-I, Ganodermenonol	in vitro, in silico	C-33A, A549	docks TNF	N/R	Yes, ARPE-19
[129]	*G. lucidum*	Ganodermanontriol (GDNT)	in vitro	MCF7, MDA-MB-231	↓ CDC20, ↓ uPA (PLAU), ↓ uPAR	N/R	N/R
[8]	None	30 lanostanoids	in silico	n/a	docks VDR ligand-binding pocket, in silico competition vs. calcitriol	N/R	N/R
[6]	None	GA-E, GA-Df, GA-XL4, mariesiic acid A	in silico	n/a	docks c-myc	N/R	N/R
[130]	*G. lucidum*	GA-A	in vitro	HepG2	↓ Bcl-2, ↓ Mcl-1, ↑ caspase-3, ↓ Cyclin D1, ↓ ERK1/2, ↓ JAK1, ↓ JAK2, ↓ STAT3, ↑ PARP1	G1 cell cycle arrest, apoptosis	N/R
[131]	*G. lucidum*	GA-Me	in vitro, in silico	HCT116, 95-D	↓ Bax, ↓ Bcl-2, ↑ caspase-3, ↓ caspase-8, ↑ caspase-9, ↓ CDK6, ↓ Cyclin E1, ↓ IFNAR1, ↓ UPS3; docks and ↓ MMP2/9	DNA fragmentation, apoptosis	N/R
[132]	None	GTs, LA-N	in vitro	HeLa	↓ 14-3-3 β/α, ↓ eIF5A, ↓ Ku80, ↓ Peroxiredoxin 2	Weak G0/G1 phase arrest, DNA damage, ↑ ROS	N/R
[133]	*G. calidophilum*	Ganoderic aldehyde A	in vitro, in silico	MCF7, MDA-MB-231, C-33A	↑ caspases, ↑ p38 (MAPK14/11), ↓ PI3K-AKT pathway; docks and ↓ PTP1B	Apoptosis	N/R
[21]	*G. calidophilum*	Ganodecalones	in vitro, in silico	HeLa, SGC-7901, K562	docks and ↓ α-glucosidase, ↓ PTP1B	N/R	N/R
[134]	*G. lucidum*	LA-B	in vitro	HepG2	↓ AP-1, ↓ c-fos, ↓ c-jun, ↓ ERK1/2, ↑ IκBα, ↓ MMP9, ↓ NF-κB	N/R	N/R
[7]	None	GS-1, GA-A, and GA-DM	in silico	n/a	↓ DNA Topoisomerase 2-beta	N/R	N/R
[135]	*G. lucidum*	GA-A, GA-C, methyl-ganoderate-A	in vitro	n/a	↓ farnesyl protein transferase	N/R	N/R
[136]	*G. lucidum*	GA-T	in vitro	HCT116	↑ IκBα, ↓ iNOS, ↓ MMP2/9, ↓ NF-κB, ↑ p53, ↓ p65 (RELA), ↓ TNF, ↓ uPA (PLAU)	N/R	N/R
[137]	*G. sichuanense (lingzhi)*	Various. Mainly GA-TN, GA-TQ, GA-TR; Ganoderols; Lucidumols	in vitro, in silico	n/a	docks and ↓ ROCK1, ↓ ROCK2	N/R	N/R
[138]	None	GA-D	in vitro, in silico	HeLa	↑ 14-3-3E, ↓ eIF5A, ↓ MAPRE1 (EB1), ↑ PRDX3 expression; docks six isoforms of 14-3-3 protein family, aminopeptidase B, annexin V; in silico interaction with HSPA70, HSP90AA1, and XPO1	G2/M cell cycle arrest, DNA fragmentation	N/R
[17]	*G. lucidum*	endertiin B	in vitro	MCF7, MDA-MB-231	↑ Bax, ↑ Bak, ↓ Bcl-2, ↓ Cyclin D1, ↑ p21 (CDKN1A), ↑ p27 (CDKN1B), ↓ PI3K-AKT pathway	G0/G1 cell cycle arrest	N/R
[139]	*G. lucidum*	GA-D	in vitro	SKOV-3	↓ ERK	↑ ROS	N/R
[140]	*G. lucidum*	Lanostane triterpenes, namely Ganoderol A	in vitro	n/a	docks and ↓ Steroid Sulfatase	N/R	N/R
[141]	*G. lucidum*	GA-Mk, GA-T, GA-T1, GA-T2	in vitro	HeLa	↑ caspase-3, ↑ caspase-9, ↓ GPX, and glutathione, ↓ SOD	↑ ROS, ↓ antioxidant capacity, ↓ ΔΨm	N/R
[142]	*G. lucidum*	GTs	in silico	n/a	docks CDK22 and PPAR-γ	Histone acetylation	N/R
[12]	None	5-FU with GA Nanoparticle	in vitro	MCF7	↓ Bcl-2, ↑ caspase-9	Apoptosis	N/R
[143]	*G. lucidum*	Lucidenic Acids A, B, C, and N	in vitro	HepG2	↓ MMP9	N/R	N/R
[144]	*G. lucidum*	Triterpene-enriched fraction	in vitro	HuH-7	↑ Cyclin B12, ↑ JNK, ↑ p38 (MAPK14/11)	G2 cell cycle arrest	N/R
[11]	*G. sichuanense (lingzhi)*	GA-S, GA-T-Q, GA-TR, Ganoderiol F, Ganodermanontriol	in vitro	n/a	Tubulin-β polymer stabilization (like paclitaxel)	N/R	N/R
[145]	None	GA-F, GA-X, GA-Y	in silico	n/a	docks MDM2	N/R	N/R

Up (↑) and down (↓) arrows indicate whether the protein, mRNA, or phenomenon increased or decreased in quantity or functionality. GA: ganoderic acid; N/R: none reported within the study; ΔΨm: mitochondrial membrane potential; ROS: reactive oxygen species.

**Table 3:** List of the 136 proteins affected in human cancer cells after treatment with *Ganoderma* triterpenoids (GTs).

Name	Description	UniProt Accession Number	Reported In Vitro	Reported In Silico
ABCB1	ATP-binding cassette subfamily B member 1	P08183	*	*
ABCC1	ATP-binding cassette subfamily C member 1	P33527	*	
ABCC2	ATP-binding cassette subfamily C member 2	Q92887	*	
ACACA	acetyl-CoA carboxylase alpha	Q13085	*	
AKT1	AKT serine/threonine kinase 1	P31749	*	
ALK	ALK receptor tyrosine kinase	Q9UM73	*	
ANXA5	annexin A5	P08758	*	*
AR	androgen receptor	P10275	*	
ATG5	autophagy-related 5	Q9H1Y0	*	
BAK1	BCL2 antagonist/killer 1	Q16611	*	
BAX	BCL2-associated X, apoptosis regulator	Q07812	*	
BCL2	BCL2 apoptosis regulator	P10415	*	
BCL2L1	BCL2-like 1	Q07817	*	
BECN1	beclin 1	Q14457	*	
CAPN2	calpain 2	P17655	*	
CAPN8	calpain 8	A6NHC0	*	
CASP3	caspase 3	P42574	*	
CASP7	caspase 7	P55210	*	
CASP8	caspase 8	Q14790	*	
CASP9	caspase 9	P55211	*	
CCNA2	cyclin A2	P20248	*	
CCNB1	cyclin B1	P14635	*	
CCND1	cyclin D1	P24385	*	
CCNE1	cyclin E1	P24864	*	
CDC20	cell division cycle 20	Q12834	*	
CDC25C	cell division cycle 25C	P30307	*	
CDK1	cyclin-dependent kinase 1	P06493	*	
CDK2	cyclin-dependent kinase 2	P24941	*	*
CDK3	cyclin-dependent kinase 3	Q00526	*	
CDK6	cyclin-dependent kinase 6	Q00534	*	
CDKN1A	cyclin-dependent kinase inhibitor 1A	P38936	*	
CDKN1B	cyclin-dependent kinase inhibitor 1B	P46527	*	
CDKN2A	cyclin-dependent kinase inhibitor 2A	P42771	*	
CTNNB1	catenin beta 1	P35222	*	*
CXCL8	C-X-C motif chemokine ligand 8	P10145	*	
CYCS	cytochrome c, somatic	P99999	*	
DDIT3	DNA damage inducible transcript 3	P35638	*	
EGFR	epidermal growth factor receptor	P00533	*	*
EIF2AK3	eukaryotic translation initiation factor 2 alpha kinase 3	Q9NZJ5	*	
EIF2S1	eukaryotic translation initiation factor 2 subunit alpha	P05198	*	
EIF5A	eukaryotic translation initiation factor 5A	P63241	*	
ESR1	estrogen receptor 1	P03372		*
FLT1	fms-related receptor tyrosine kinase 1	P17948	*	
FNTA	farnesyltransferase, CAAX box, subunit alpha	P49354	*	
FOS	Fos proto-oncogene, AP-1 transcription factor subunit	P01100	*	
FZD4	frizzled class receptor 4	Q9ULV1	*	
GAA	alpha glucosidase	P10253	*	*
GPX2	glutathione peroxidase 1	P18283	*	
GSK3B	glycogen synthase kinase 3 beta	P49841	*	
H2AX	H2A.X variant histone	P16104	*	
HLA-DRA	major histocompatibility complex, class II, DR alpha	P01903	*	
HNRNPK	heterogeneous nuclear ribonucleoprotein K	P61978	*	
HSP90AA1	heat shock protein 90 alpha family class A member 1	P07900		*
HSPA1A	heat shock protein family A (Hsp70) member 1A	P0DMV8	*	*
HSPA4	heat shock protein family A (Hsp70) member 4	P34932	*	
HSPA5	heat shock protein family A (Hsp70) member 5	P11021	*	
IFNAR1	interferon alpha and beta receptor subunit 1	P17181	*	
IGF1R	insulin-like growth factor 1 receptor	P08069		*
IKBKG	inhibitor of nuclear factor kappa B kinase regulatory subunit gamma	Q9Y6K9		*
IL1R1	interleukin 1 receptor type 1	P14778	*	*
IL25	interleukin 25	Q9H293	*	
IL6	interleukin 6	P05231	*	
INSR	insulin receptor	P06213		*
JAK1	Janus kinase 1	P23458	*	
JAK2	Janus kinase 2	O60674	*	
JUN	Jun proto-oncogene, AP-1 transcription factor subunit	P05412	*	
KDR	kinase insert domain receptor	P35968	*	
LAMP2	lysosomal-associated membrane protein 2	P13473	*	
LRP5	LDL receptor-related protein 5	O75197	*	
MAP1LC3A	microtubule-associated protein 1 light chain 3 alpha	Q9H492	*	
MAP1LC3B	microtubule-associated protein 1 light chain 3 beta	Q9GZQ8	*	
MAPK1	mitogen-activated protein kinase 1	P28482	*	
MAPK11	mitogen-activated protein kinase 11	Q15759	*	
MAPK14	mitogen-activated protein kinase 14	Q16539	*	
MAPK3	mitogen-activated protein kinase 3	P27361	*	
MAPK8	mitogen-activated protein kinase 8	P45983	*	
MAPK9	mitogen-activated protein kinase 9	P45984	*	
MAPRE1	microtubule-associated protein RP/EB family member 1	Q15691	*	
MCL1	MCL1 apoptosis regulator, BCL2 family member	Q07820	*	
MDM2	MDM2 proto-oncogene	Q00987	*	*
MMP2	matrix metallopeptidase 2	P08253	*	*
MMP9	matrix metallopeptidase 9	P14780	*	*
MTOR	mechanistic target of rapamycin kinase	P42345	*	
MYC	MYC proto-oncogene, bHLH transcription factor	P01106	*	*
NFE2L2	Nuclear factor erythroid 2-related factor 2	Q16236	*	*
NFKB1	nuclear factor kappa B subunit 1	P19838	*	
NFKBIA	NFKB inhibitor alpha	P25963	*	
NOS2	nitric oxide synthase 2	P35228	*	
NOTCH1	notch receptor 1	P46531	*	*
NUCB1	nucleobindin 1	Q02818	*	
PARK7	Parkinsonism-associated deglycase	Q99497	*	
PARP1	poly(ADP-ribose) polymerase 1	P09874	*	
PGGT1B	protein geranylgeranyltransferase type I subunit beta	P53609	*	
PIK3CA	phosphatidylinositol-4,5-bisphosphate 3-kinase catalytic subunit alpha	P42336	*	*
PLAU	plasminogen activator, urokinase	P00749	*	
PPARG	peroxisome proliferator-activated receptor gamma	P37231		*
PPID	peptidylprolyl isomerase D	Q08752	*	
PPP2R1A	protein phosphatase 2 scaffold subunit A alpha	P30153	*	
PRDX2	peroxiredoxin 2	P32119	*	
PRDX3	peroxiredoxin 3	P30048	*	
PRKAA1	protein kinase AMP-activated catalytic subunit alpha 1	Q13131	*	
PRKAA2	protein kinase AMP-activated catalytic subunit alpha 2	P54646	*	
PTPN1	protein tyrosine phosphatase non-receptor type 1	P18031	*	*
RCN1	reticulocalbin 1	Q15293	*	
RELA	RELA proto-oncogene, NF-κB subunit	Q04206	*	
RNPEP	arginyl aminopeptidase	Q9H4A4	*	*
ROCK1	Rho-associated coiled-coil-containing protein kinase 1	Q13464	*	*
ROCK2	Rho-associated coiled-coil-containing protein kinase 2	O75116	*	*
RPS6KB1	ribosomal protein S6 kinase B1	P23443	*	
SIRT3	sirtuin 3	Q9NTG7	*	
SLC2A1	solute carrier family 2 member 1	P11166	*	*
SLC2A3	solute carrier family 2 member 3	P11169	*	*
SOD1	superoxide dismutase 1	P00441	*	
SQSTM1	sequestosome 1	Q13501	*	
SRD5A1	steroid 5 alpha-reductase 1	P18405	*	
STAT1	signal transducer and activator of transcription 1	P42224		*
STAT3	signal transducer and activator of transcription 3	P40763	*	*
STS	steroid sulfatase	P08842	*	*
TNF	tumour necrosis factor	P01375	*	*
TNFRSF10B	TNF receptor superfamily member 10b	O14763	*	*
TOP1	DNA topoisomerase I	P11387	*	
TOP2A	DNA topoisomerase II alpha	P11388	*	*
TP53	tumour protein p53	P04637	*	
TP53BP1	tumour protein p53 binding protein 1	Q12888	*	
TSPAN12	tetraspanin 12	O95859	*	
TUBA1B	tubulin alpha 1b	P68363	*	
UBQLN2	ubiquilin 2	Q9UHD9	*	
ULK1	unc-51-like autophagy activating kinase 1	O75385	*	
USP2	ubiquitin-specific peptidase 2	O75604	*	
VDR	vitamin D receptor	P11473		*
VEGFA	vascular endothelial growth factor A	P15692	*	*
WNT5A	Wnt family member 5A	P41221	*	
XBP1	X-box binding protein 1	P17861	*	
XPO1	exportin 1	O14980	*	
XRCC5	X-ray repair cross-complementing 5	P13010	*	
YWHAB	14-3-3 beta/alpha	P31946	*	*

An asterisk (*) denotes the type of assay used to detect the target.

## Data Availability

The data are contained in the article and Supplementary Materials.

## Supplementary Materials

The following supporting information can be downloaded at: https://www.mdpi.com/article/10.3390/ph18111641/s1, Figure S1. Top 10 nodes with the highest betweenness centrality values (bottlenecks) after topological analysis using CytoHubba plugin. The redness of the colour represents the degree of connectivity. File S1. sd1: Search Strategy; File S2. sd2: Excluded articles and reasons for exclusion; File S3. sd3: Molecular participants of ICD; File S4. sd4: Cytoscape network containing the GTs molecular targets; File S5. sd5: Cytoscape network containing GTs-ICD intersecting molecular targets; File S6. sd6: Enrichment analyses raw data; File S7. sd7: Protein interaction network and enrichment analysis of GTs in vitro-only data.

## Abbreviations

The following abbreviations are used in the manuscript:

ANXA1	annexin A1
ATP	adenosine triphosphate
CALR	calreticulin
DAMP	damage-associated molecular pattern
DC	dendritic cell
ERS	endoplasmic reticulum stress
GA	ganoderic acid
GO:BP	gene ontology: biological process
GT	*Ganoderma* triterpenoid
HLA	human leukocyte antigen
HMGB1	high-mobility group box 1 protein
HSP70/90	heat shock protein 70/90
KEGG	Kyoto Encyclopaedia of Genes and Genomes
MHC	major histocompatibility complex
PAMP	pathogen-associated molecular pattern
PRR	pattern recognition receptor
ROS	reactive oxygen species
ΔΨm	mitochondrial membrane potential
