# Supplementary material for: Can Ganoderma Triterpenoids Exert Immunogenic Cell Death in Human Cancer Cells? A Systematic Review and Protein Network Analysis
Source: Pharmaceuticals (Basel). 2025 Oct 30;18(11):1641. doi: 10.3390/ph18111641 (PMC12655067; doi:10.3390/ph18111641)
Supplement: Supplementary file 1 [file pharmaceuticals-18-01641-s001.zip › renamed supplementary material/Figure S1.odt]

B

 

Figure S1. Top 10 nodes with the highest betweenness centrality values (bottlenecks) after topological analysis using CytoHubba plugin, the redness of the colour represents the amount of degree connectivity.
