# Supplementary material for: Can Ganoderma Triterpenoids Exert Immunogenic Cell Death in Human Cancer Cells? A Systematic Review and Protein Network Analysis
Source: Pharmaceuticals (Basel). 2025 Oct 30;18(11):1641. doi: 10.3390/ph18111641 (PMC12655067; doi:10.3390/ph18111641)
Supplement: Supplementary file 1 [file pharmaceuticals-18-01641-s001.zip › renamed supplementary material/sd1_-_search_strategy.odt]

Systematic review

- WOS

((TI=(Cancer) OR AB=(Cancer) OR WC=(Oncology))

AND

(AB=(("Ganoderic acid") OR ("Ganoderic acids") OR ("Lucidenic acid") OR ("Lucidenic acids")) OR (TS=(Ganoderma) AND TS=(Lanostane OR Lanostanoid)))) NOT (ALL=(mouse OR mice OR rat OR rats OR murine))

106 found

=====

- PubMed GA and LA

(Cancer) AND ("Ganoderic acid" OR "Lucidenic acid" OR "Ganoderic acids" OR "Lucidenic acids") NOT Review[pt] NOT (mice OR rat) NOT "Antigens, Viral"[Mesh]

73 found

=====

- PubMed Lanostanoid found

(Cancer) AND (Ganoderma[tiab]) AND ((Lanostane[tiab]) OR (Lanostanoid[tiab])) NOT Review[pt] NOT (mice OR rat) NOT "Antigens, Viral"[Mesh]

31 found

Total PubMed = 104, Final PubMed = 98

Ergo, PubMed duplicates = 6

PubMed = 98, WOS = 106

Total found = 204

Final total = 129

Ergo, duplicates between WOS and PubMed = 75
